# The effect of control strategies to reduce social mixing on outcomes of the COVID-19 epidemic in Wuhan, China: a modelling study

**DOI:** 10.1016/S2468-2667(20)30073-6

**Published:** 2020-03-25

**Authors:** Kiesha Prem, Yang Liu, Timothy W Russell, Adam J Kucharski, Rosalind M Eggo, Nicholas Davies, Centre for the Mathematical Modelling of Infectious Diseases COVID-19 Working Group, Stefan Flasche, Samuel Clifford, Carl A B Pearson, James D Munday, Sam Abbott, Hamish Gibbs, Alicia Rosello, Billy J Quilty, Thibaut Jombart, Fiona Sun, Charlie Diamond, Amy Gimma, Kevin van Zandvoort, Sebastian Funk, Christopher I Jarvis, W John Edmunds, Nikos I Bosse, Joel Hellewell, Mark Jit, Petra Klepac

**Affiliations:** a Centre for Mathematical Modelling of Infectious Diseases, Department of Infectious Disease Epidemiology, London School of Hygiene & Tropical Medicine, London, UK

## Abstract

**Background:**

In December, 2019, severe acute respiratory syndrome coronavirus 2 (SARS-CoV-2), a novel coronavirus, emerged in Wuhan, China. Since then, the city of Wuhan has taken unprecedented measures in response to the outbreak, including extended school and workplace closures. We aimed to estimate the effects of physical distancing measures on the progression of the COVID-19 epidemic, hoping to provide some insights for the rest of the world.

**Methods:**

To examine how changes in population mixing have affected outbreak progression in Wuhan, we used synthetic location-specific contact patterns in Wuhan and adapted these in the presence of school closures, extended workplace closures, and a reduction in mixing in the general community. Using these matrices and the latest estimates of the epidemiological parameters of the Wuhan outbreak, we simulated the ongoing trajectory of an outbreak in Wuhan using an age-structured susceptible-exposed-infected-removed (SEIR) model for several physical distancing measures. We fitted the latest estimates of epidemic parameters from a transmission model to data on local and internationally exported cases from Wuhan in an age-structured epidemic framework and investigated the age distribution of cases. We also simulated lifting of the control measures by allowing people to return to work in a phased-in way and looked at the effects of returning to work at different stages of the underlying outbreak (at the beginning of March or April).

**Findings:**

Our projections show that physical distancing measures were most effective if the staggered return to work was at the beginning of April; this reduced the median number of infections by more than 92% (IQR 66–97) and 24% (13–90) in mid-2020 and end-2020, respectively. There are benefits to sustaining these measures until April in terms of delaying and reducing the height of the peak, median epidemic size at end-2020, and affording health-care systems more time to expand and respond. However, the modelled effects of physical distancing measures vary by the duration of infectiousness and the role school children have in the epidemic.

**Interpretation:**

Restrictions on activities in Wuhan, if maintained until April, would probably help to delay the epidemic peak. Our projections suggest that premature and sudden lifting of interventions could lead to an earlier secondary peak, which could be flattened by relaxing the interventions gradually. However, there are limitations to our analysis, including large uncertainties around estimates of *R*_0_ and the duration of infectiousness.

**Funding:**

Bill & Melinda Gates Foundation, National Institute for Health Research, Wellcome Trust, and Health Data Research UK.

## Introduction

Severe acute respiratory syndrome coronavirus 2 (SARS-CoV-2), a novel coronavirus, emerged in the city of Wuhan, Hubei, China, in early December, 2019.^1^, ^2^ Since then, the local and national governments have taken unprecedented measures in response to the coronavirus disease 2019 (COVID-19) outbreak caused by SARS-CoV-2.^3^ Exit screening of passengers was shortly followed by travel restrictions in Wuhan on Jan 23, 2020, halting all means of unauthorised travel into and out of the city. Similar control measures were extended to the entire province of Hubei by Jan 26, 2020.^3^ Non-pharmaceutical physical distancing interventions, such as extended school closures and workplace distancing, were introduced to reduce the impact of the COVID-19 outbreak in Wuhan.^4^ Within the city, schools remained closed, Lunar New Year holidays were extended so that people stayed away from their workplaces, and the local government promoted physical distancing and encouraged residents to avoid crowded places. These measures greatly changed age-specific mixing patterns within the population in previous outbreak response efforts for other respiratory infectious diseases.^5^, ^6^ Although travel restrictions undoubtedly had a role in reducing exportations of infections outside Wuhan and delayed the onset of outbreaks in other regions,^7^, ^8^ changes in mixing patterns affected the trajectory of the outbreak within Wuhan itself. To estimate the effects of physical distancing measures on the progression of the COVID-19 epidemic, we look at Wuhan, hoping to provide some insights for the rest of the world.

**Research in context**

**Evidence before this study**

Severe acute respiratory syndrome coronavirus 2 (SARS-CoV-2) emerged in Wuhan, China in late 2019. In mid-January, 2020, schools and workplaces closed as part of the Lunar New Year holidays. These closures were then extended to prevent SARS-CoV-2 spread. The intended effect of such physical distancing measures was to reduce person-to-person contact, which spreads infectious diseases. Epidemic parameters, such as time-dependent reproduction numbers governing SARS-CoV-2 transmission in Wuhan, have been estimated based on local and internationally exported cases. The frequency of contacts in different age groups and locations (schools, workplaces, households, and others) in China has also been previously estimated. We searched PubMed and medRxiv for studies published in English up to March 7, 2020, with the terms “coronavirus AND (school OR work) AND (Wuhan OR Hubei)” and identified 108 and 130 results, respectively. However, to our knowledge, no published article has reported use of location-specific transmission models that consider the impacts of school or workplace closures to study the spread of SARS-CoV-2 in Wuhan.

**Added value of this study**

We built an age-specific and location-specific transmission model to assess progression of the Wuhan outbreak under different scenarios of school and workplace closure. We found that changes to contact patterns are likely to have substantially delayed the epidemic peak and reduced the number of coronavirus disease 2019 (COVID-19) cases in Wuhan. If these restrictions are lifted in March, 2020, a second peak of cases might occur in late August, 2020. Such a peak could be delayed by 2 months if the restrictions were relaxed a month later, in April, 2020.

**Implications of all the available evidence**

The measures put in place to reduce contacts in school and work are helping to control the COVID-19 outbreak by affording health-care systems time to expand and respond. Authorities need to carefully consider epidemiological and modelling evidence before lifting these measures to mitigate the impact of a second peak in cases.

Person-to-person transmission is mostly driven by who interacts with whom,^9^, ^10^ which can vary by age and location of the contact (ie, school, work, home, and community). Under the context of a large-scale ongoing outbreak, contact patterns would drastically shift from their baseline conditions. In the COVID-19 outbreak in Wuhan, physical distancing measures, including but not limited to school and workplace closures and health promotions that encourage the general public to avoid crowded places, are designed to drastically shift social mixing patterns and are often used in epidemic settings.^4^ Although contact patterns can be inferred from reported social contact data that include information on which setting the contact took place in, such studies are often focused on high-income countries,^11^ or particular high-density areas.^12^ This limitation can be addressed by quantifying contact patterns in the home, school, work, and other locations across a range of countries based on available information from household-level data and local population demographic structures.^13^

To examine how these changes in population mixing have affected the outbreak progression, we used synthetic location-specific contact patterns in Wuhan and adapted these in the presence of school closures, extended workplace closures, and reduction in mixing in the general community. Using these matrices and the latest estimates of the epidemiological parameters of the Wuhan outbreak,^1^, ^9^, ^14^, ^15^, ^16^ we simulated the ongoing trajectory of an outbreak in Wuhan using an age-structured susceptible-exposed-infected-removed (SEIR) model^17^, ^18^ for several physical distancing measures.

## Methods

### SEIR model

We simulated the outbreak in Wuhan using a deterministic stage-structured SEIR model over a 1 year period, during which the modelled outbreak peters out. An implication of this approach is that all demographic changes in the population (ie, births, deaths, and ageing) are ignored.

**Figure 1:**
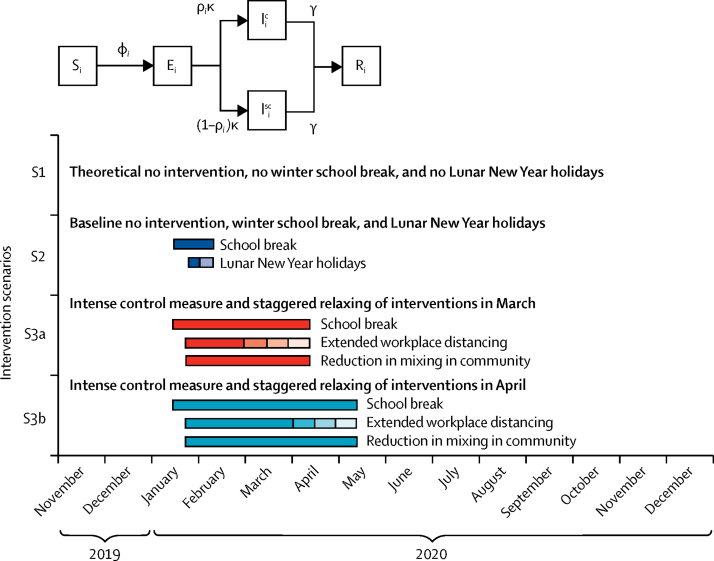
Age-structured SEIR model and details of the modelled physical distancing interventions According to infection status, we divided the population into susceptible (S), exposed (E), infected (I), and removed (R) individuals. An infected individual in an age group can be clinical (I^c^) or subclinical (I^sc^), and ρ_i_ refers to the probability that an individual is symptomatic or clinical. The age-specific mixing patterns of individuals in age group *i*, C_i,j_, alter their likelihood of being exposed to the virus given a certain number of infected individuals in the population. Younger individuals are more likely to be asymptomatic and less infectious, ie, subclinical. When ρ_i_=0 for all *i*, the model simplifies to a standard SEIR. The force of infection φ_i,t_ is given by 1–(βΣ_j_C_i,j_*I*^C^_j,t_+αβΣ_j_C_i,j_*I*^SC^_j,t_), where β is the transmission rate and α is the proportion of transmission that resulted from a subclinical individual. SEIR= susceptible-exposed-infected-removed.

We divided the population according to the infection status into susceptible (S), exposed (E), infected (I), and removed (R) individuals, and according to age into 5-year bands until age 70 years and a single category aged 75 and older (resulting in 16 age categories). Susceptible individuals might acquire the infection at a given rate when they come in contact with an infectious person and enter the exposed disease state before they become infectious and later either recover or die. We assumed Wuhan to be a closed system with a constant population size of 11 million (ie, S + E + I + R=11 million) throughout the course of this epidemic. We used the SEIR model presented in figure 1. The age-specific mixing patterns of individuals in age group *i* alter their likelihood of being exposed to the virus given a certain number of infectious people in the population. Additionally, we incorporated contributions of asymptomatic and subclinical cases; however, the question of whether such individuals are able to transmit infection remains unresolved at the time of writing, although evidence suggests that they are likely to.^19^ We also considered a scenario in which we assumed that younger individuals are more likely to be asymptomatic (or subclinical) and less infectious than older individuals.^20^, ^21^

For a given age group *i*, epidemic transitions can be described by

$$S_{i,t+1}=S_{i,t}-\beta S_{i,t}\sum_{j=1}^{n}C_{i,j}I_{j,t}^{c}-\alpha \beta_{i,t}\sum_{j=1}^{n}C_{i,j}I_{j,t}^{sc}$$

$$E_{i,t+1}=\left(1-\kappa \right)E_{i,t}+\beta S_{i,t}\sum_{j=1}^{n}C_{i,j}I_{j,t}^{c}+\alpha \beta S_{i,t}\sum_{j=1}^{n}C_{i,j}I_{j,t}^{SC}$$

$$I_{j,t+1}=\rho_{i}\kappa E_{i,t}+(1-\gamma )I_{j,t}^{c}$$

$$I_{j,t+1}=(1-\rho_{i})\kappa E_{i,t}+(1-\gamma )I_{j,t}^{sc}$$

$$R_{i,t+1}=R_{i,t}+\gamma I_{j,t+1}^{c}+\gamma I_{j,t+1}^{sc}$$

Where β is the transmission rate (scaled to the right value of *R*_0_), C_i,j_ describe the contacts of age group *j* made by age group *i*, κ=1-exp(–1/*d*_L_) is the daily probability of an exposed individual becoming infectious (with *d*_L_ being the average incubation period), and γ=1–exp(–1/*d*_I_) is the daily probability that an infected individual recovers when the average duration of infection is *d*_I_. We also incorporated contributions of asymptomatic and subclinical cases, 1–ρ_i_ denotes the probability of an infected case being asymptomatic or subclinical. We assumed that younger individuals are more likely to be asymptomatic (or subclinical) and less infectious (proportion of infectiousness compared to *I*^c^, α).

Using parameters from the literature as presented in the table, we simulated the outbreak. We assumed the mean incubation period and mean infectious period to be 6·4 days^16^ and 3 days or 7 days,^22^ respectively. Each simulation started with 200 or 2000 infectious individuals *I*_0_,^15^ with the rest of the population being in the susceptible state. We explored the uncertainty in the model by drawing *R*_0_ values uniformly from the 95% CI from the posterior of the *R*_0_ distribution from the semi-mechanistic model by Kucharski and colleagues (appendix p 2).^14^

**Table:** Parameters of the susceptible-exposed-infected-removed model

	**Values**	**References**
Basic reproduction number, *R*_0_	2·2 (1·6–3·0)*	Kucharski et al^14^
Average incubation period, *d*_L_	6·4 days	Backer et al^16^
Average duration of infection, *d*_I_	3 days or 7 days	Woelfel et al^22^
Initial number of infected, *I*_0_	200 or 2000	Abbott et al^15^
Pr (infected case is clinical), ρ_i_	0 or 0·4, for *i*≤4	Bi et al^20^
Pr (infected case is clinical), ρ_i_	0 or 0·8, for *i*>4	Davies^21^
Pr (infection acquired from subclinical), α	0·25	Liu et al^19^

* Data are median (IQR). Pr represents the probability of an event. The parameters *d*_L_ and *d*_I_ represent the mean incubation period and duration of infectiousness, respectively.

### Social mixing and interventions

**Figure 2:**
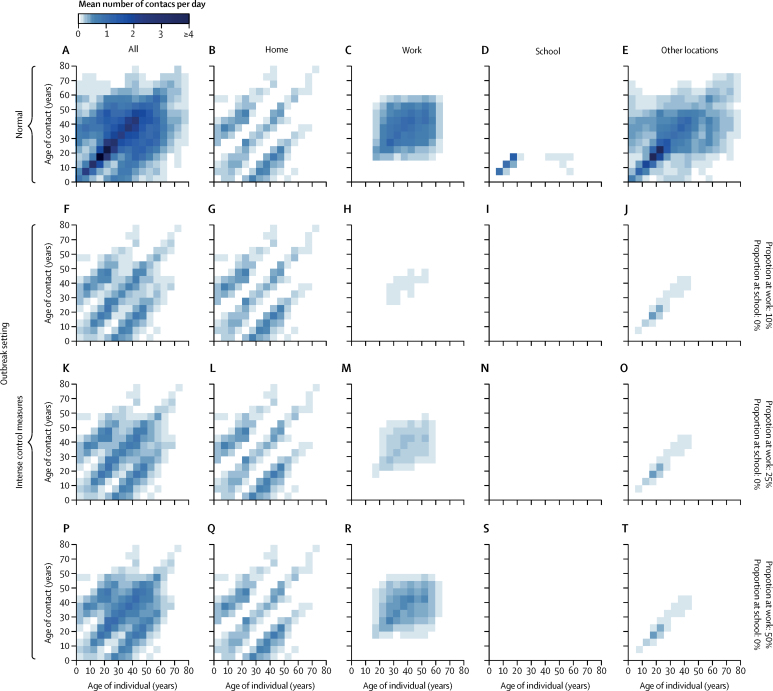
Synthetic age-specific and location-specific contact matrices for China under various physical distancing scenarios during the intense control period for China Synthetic age-specific contact patterns across all locations, at home, in the workplace, in school, and at other locations during normal circumstances (ie, under no intervention) are presented in panels A to E. Age-specific and location-specific contact matrices under the various physical distancing interventions are presented in panels F to T. Darker colour intensities indicate higher proclivity of making the age-specific contact.

Social mixing patterns vary across locations, including households, workplaces, schools, and other locations. Therefore, we used the method set out by Prem and colleagues,^13^ which accounts for these differences and obtains the location-specific contact matrices (C) for different scenarios. In a normal setting, contacts made at all of these locations contribute to the overall mixing pattern in a population, so we summed contacts across the different locations to obtain our baseline contact pattern in the population before the outbreak (figure 2; appendix pp 1–2). In an outbreak setting, different intervention strategies are aimed at reducing social mixing in different contexts to lower the overall transmission in the population. To simulate the effects of interventions aimed at reducing social mixing, we created synthetic contact matrices for each intervention scenario from these building block matrices.

We considered the following three scenarios: first scenario, theoretical: assumed no change to social mixing patterns at all location types, no school term break, and no Lunar New Year holidays; second scenario, no interventions, winter school break in Wuhan, and Lunar New Year holidays: assumed no physical distancing control measures, school-going individuals did not have any contacts at school because of school holidays from Jan 15, to Feb 10, 2020, and 10% and later 75% of workforce would be working during the holidays from Jan 25, to Jan 31, 2020, and from Feb 1, to Feb 10, 2020, respectively; and third scenario, intense control measures in Wuhan to contain the outbreak: assumed school closure and about 10% of workforce (eg, health-care personnel, police, and other essential government staff) would be working even during the control measures (Figure 1, Figure 2). For the third scenario, we modelled the effect of the intense control measures ending at the beginning of March or April, and we allowed for a staggered return to work while the school remained closed (ie, 25% of the workforce working in weeks one and two, 50% of the workforce working in weeks three and four, and 100% of the workforce working and school resuming (figure 2).^3^, ^23^, ^24^

Analyses and model building were done in R version 3.6.2.

### Role of the funding source

The funder of the study had no role in study design, data collection, data analysis, data interpretation, or writing of the report. The corresponding author had full access to all the data in the study and had final responsibility for the decision to submit for publication.

## Results

**Figure 3:**
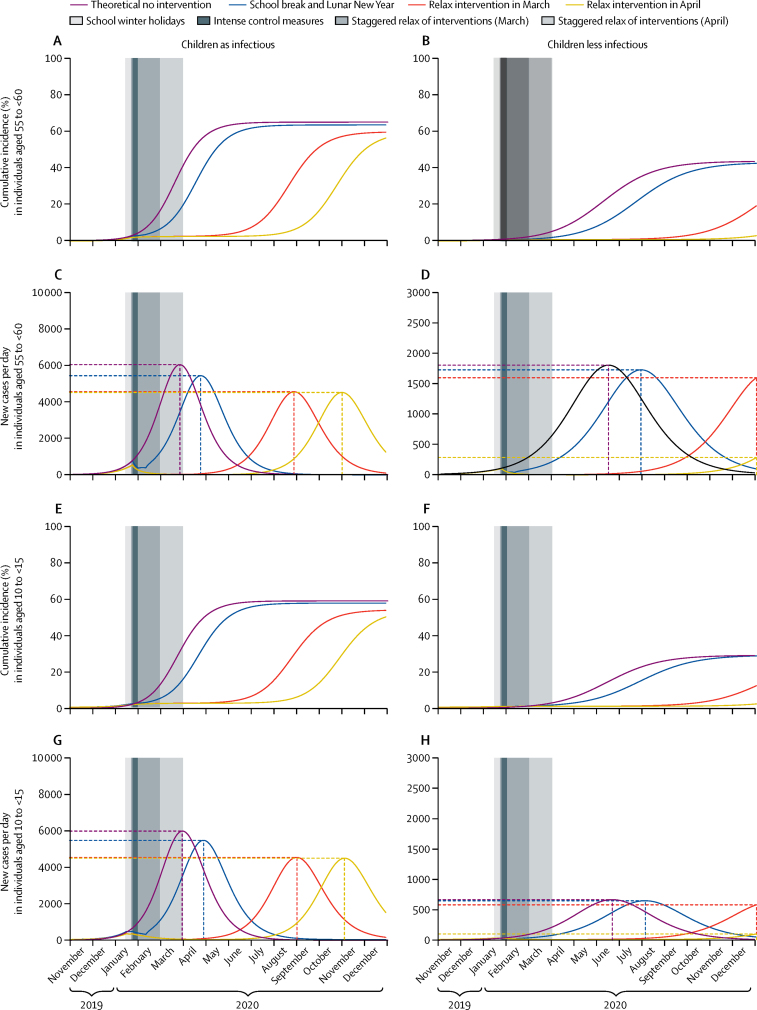
Effects of different intervention strategies on cumulative incidence and new cases per day among individuals aged 55 to <60 years (A to D) and 10 to <15 years (E to H) from late 2019 to end-2020

Our simulations showed that control measures aimed at reducing social mixing in the population can be effective in reducing the magnitude and delaying the peak of the COVID-19 outbreak. For different control measures among individuals aged 55 to <60 years and 10 to <15 years, the standard school winter break and holidays for the Lunar New Year would have had little effect on progression of the outbreak had schools and workplaces reopened as normal (figure 3).

**Figure 4:**
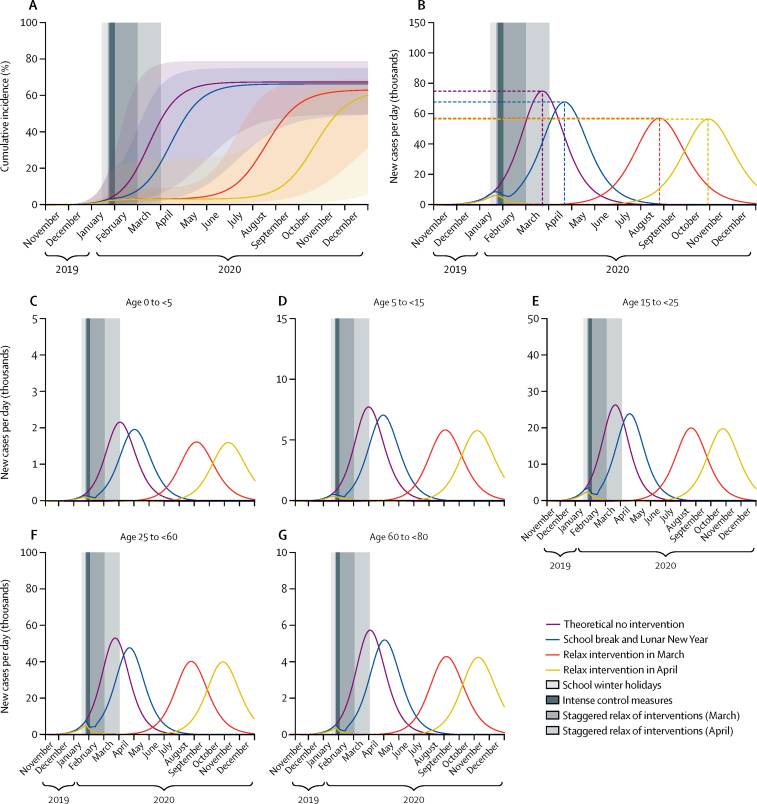
Effects of different physical distancing measures on cumulative incidence (A) and new cases per day (B), and age-specific incidence per day (C to G) from late 2019 to end-2020 Results depicted here assume an infectious period of 7 days. Median cumulative incidence, incident cases per day, and age-specific incidence per day are represented as solid lines. Shaded areas around the coloured lines in panel A represent the IQR.

**Figure 5:**
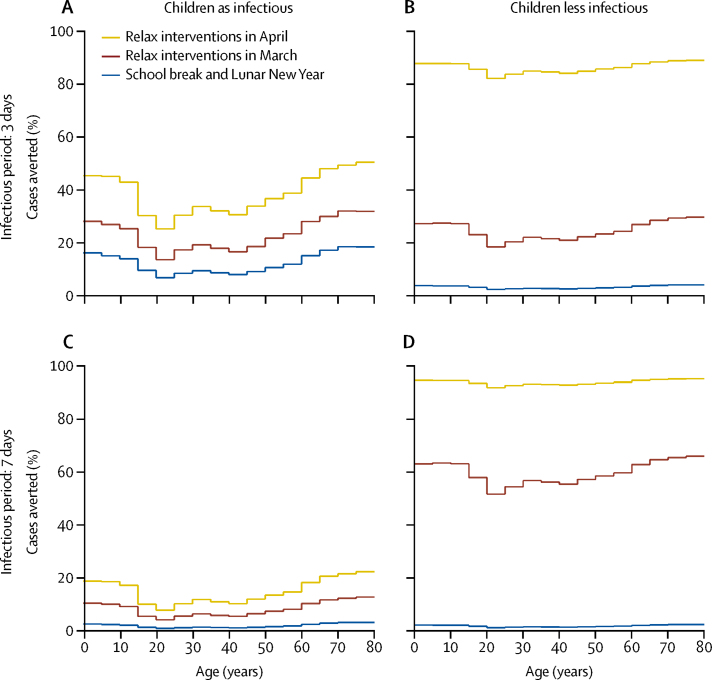
Modelled proportion of number of infections averted by end-2020 by age for different physical distancing measures, assuming the duration of infectiousness to be 3 days (A, B) or 7 days (C, D) The additional proportions of cases averted (compared with no intervention) are presented across age and by the different physical distancing measures.

We present the median cumulative incidence, incident cases per day, and age-specific incidence per day of 200 simulated outbreaks (figure 4). Intense control measures of prolonged school closure and work holidays reduced the cumulative infections by end-2020 and peak incidence, while also delaying the peak of the outbreak (figure 4). Our model suggests that the effects of these physical distancing strategies vary across age categories; the reduction in incidence is highest among school children and older individuals and lowest among working-age adults (figure 4; figure 5).

Physical distancing measures were most effective if the staggered return to work was at the beginning of April; this reduced the median number of infections by more than 92% (IQR 66–97) and 24% (13–90) in mid-2020 and end-2020, respectively (figure 5; appendix p 3), should the disease have a longer duration of infectiousness, and reduced the magnitude and delayed peak incidence across all age categories (figure 4), which could have had further beneficial impact by relieving the pressure on the health-care system in the immediate few months after the outbreak began. Uncertainty in *R*_0_ values has a large effect on the timing of the epidemic peak and final size of the outbreak (figure 4).

The modelled effects of the intense control measures of prolonged school closure and work holidays vary by the duration of infectiousness. If the disease had a short infectious period (3 days), then our model suggests that relaxing physical distancing interventions in March (figure 5; appendix p 4) could avert around 30% of cases in school children and older individuals. Fewer cases could be averted by end-2020 should the disease have a longer duration of infectiousness (eg, 7 days; figure 5); physical distancing interventions would need to be relaxed a month later (in April) to observe a larger effect. If children were less infectious, lifting physical distancing interventions in April instead of March could engender additional health benefits (figure 5; appendix pp 5–6).

## Discussion

COVID-19, a contact-transmissible infectious disease, is thought to spread through a population via direct contact between individuals.^1^, ^9^, ^10^ Outbreak control measures aimed at reducing the amount of mixing in the population have the potential to delay the peak and reduce the final size of the epidemic. To evaluate the effect of location-specific physical distancing measures—such as extended school closures and interventions in workplaces—on the timing and magnitude of the peak and the final size of the epidemic, we accounted for these heterogeneities in contact networks in our model. We simulated outbreaks and modelled the interventions by scaling down the appropriate component of the contact mixing matrices for China.

Mathematical models can help us understand how SARS-CoV-2 could spread across the population and inform control measures that might mitigate future transmission.^25^, ^26^ We simulated the trajectory of the ongoing outbreak of COVID-19 in Wuhan using an age-structured SEIR model.^17^, ^18^ As individuals' mixing patterns are non-random, they influence the transmission dynamics of the disease.^11^ Models that assess the effectiveness of physical distancing interventions, such as school closure, need to account for social structures and heterogeneities in mixing of individuals.^27^, ^28^, ^29^, ^30^, ^31^ In our model, we incorporated changes to age-specific and location-specific social mixing patterns to estimate the effects of location-specific physical distancing interventions in curtailing the spread of the outbreak. The measures put in place to reduce contacts at schools and workplaces are helping control the outbreak by providing the health-care system with the time and opportunity to expand and respond. Consequently, if these restrictions are lifted prematurely, while there are still enough susceptible people to keep the *R*_e_>1 once contacts increase, the number of infections would increase. Realistically, interventions are lifted slowly, partly as an attempt to avoid a sharp increase in infection, but also for logistical and practical reasons. Therefore, we simulated lifting the interventions in a staggered fashion.

Evidence of the effects of various physical distancing measures on containing the outbreak are scarce and little is known about the behavioural changes of individuals over time, either during an outbreak or otherwise. Therefore, to model the effects of the physical distancing measures implemented in Wuhan, we assumed the effect that certain types of physical distancing have on age-specific and location-specific contact rates.

Much is unknown about the true age-specific susceptibility and transmissibility of COVID-19. Therefore, we assumed no heterogeneity in susceptibility between children. Furthermore, for simplicity, we assumed that children and adults were equally transmissible, other than the differences in their contact rates (subclinical children could be more infectious than subclinical adults; appendix pp 5–6). Similar to an influenza-like pathogen, our model suggests that interactions between school children and older individuals in the population have important public health implications, as children might have high infection rates but the elderly are more vulnerable to severe infections, with potentially fatal outcomes.^32^, ^33^ However, unlike models built for pandemic or seasonal flu, we accounted for the lack of population immunity to SARS-CoV-2.

This study describes a mathematical model that quantifies the potential impacts of physical distancing policies, relying on Wuhan as a case study. Epidemiological investigations during the WHO-China Joint Mission on COVID-19 found many infections clustered around households.^34^ Extreme physical distancing measures, including school closures, workplace closures, and avoidance of any public gatherings all at once can push the transmission to households, leading to increased clustering of household cases.^5^ As households are not explicitly included in the model, we did not consider heterogeneity and clustering of household transmission. Distinguishing between repeated and new contacts is important for disease propagation in contact network models;^35^, ^36^ more sophisticated methods that account for temporal presence within the household^37^ would be needed to characterise higher degrees of contact. Looking at limitations of our study, our compartmental model does not capture individual-level heterogeneity in contacts, which could be important in super-spreading events, particularly early in an epidemic. Combined with nosocomial infections, the risk of COVID-19 infection is potentially amplified with close contact between confirmed cases and health-care workers. However, the compartmental model we present is not equipped to explicitly consider transmission within health-care institutions and households. More complex models, such as individual-based models with familial and health-care structures, should be explored. Nosocomial infection risk among health-care workers and patients has been identified as a research gap to be prioritised in the next few months by WHO.

A key parameter is the basic reproduction number (*R*_0_), which determines how fast SARS-CoV-2 can spread through the population during the early stages of the outbreak. This is an inherently difficult parameter to estimate, since the true number of cases that can transmit infection at a given time is unknown (reported cases are likely to be just a small fraction of true cases) and probably varies over time (because of different interventions being introduced and population behaviour changing in response to the epidemic). In our analysis, we used an existing model that inferred time-dependent *R*_e_ based on the growth of reported cases in Wuhan and the number of exported cases outside China originating from Wuhan.^14^ We acknowledge that the underlying reproduction number in Wuhan could have been larger than that used in our study. However, other studies of early SARS-CoV-2 transmission dynamics in Wuhan, using different methods, arrived at the same estimate with similar ranges.^1^, ^9^

Although the precise effects of interventions might vary by country and different estimates of key parameters, our model highlights the usefulness of physical distancing interventions and the need to carefully calibrate their lifting to avoid second and subsequent waves of a COVID-19 epidemic. Areas of China outside Hubei and other east or southeast Asian regions have managed to avert a major outbreak locally and delayed the peak of the epidemic, without resorting to Hubei's extreme measures.^38^ Policy makers are advised to reapportion their resources to focus on mitigating the effects of potentially soon-to-be overwhelmed health systems.^39^

Non-physical distancing factors play a part in mitigating potential spikes in cases, especially when physical distancing measures are relaxed. The effects of seasonality on SARS-CoV-2 are difficult to predict without long time series; supporting evidence for the link between climate and COVID-19 has been largely anecdotal and based on spread in different settings and such analyses are subject to confounding.^40^, ^41^ Consequently, we have not incorporated climatic factors into our mathematical model. Future research should be directed towards understanding the potential seasonality of COVID-19 and the climatic factors that could affect its transmission dynamics. Other innovations, such as the rapid expansion of hospital capacity and testing capabilities, would shorten diagnostic and health system delays,^3^, ^38^, ^39^ thus reducing effective interactions between infectious and susceptible individuals and interrupting transmission. Effective vaccines^42^ and antivirals^43^ that are being developed could counteract this global public health threat. The extent to which these strategies can detect cases earlier and isolate infectious individuals from the susceptible pool or protect against infection is less well-understood, hence necessitating further evaluation.

Combined physical distancing and travel restrictions have aided in lowering the transmission of COVID-19 over the course of the ongoing outbreak in Wuhan.^8^, ^44^, ^45^ Evidence for this drop in transmission can be gleaned from the time-varying estimates of the reproduction number^14^ or observing that the turnover of the epidemic has occurred far before depletion of susceptible individuals, indicating the effects of the implemented measures. It is difficult to quantify whether physical distancing alone is responsible for the drop in cases, especially during the ongoing epidemic. Therefore, we took a broad view of this question, making assumptions about the results of certain forms of physical distancing and measuring the effects somewhat qualitatively. However, to some extent, physical distancing has resulted in both a shorter epidemic and a lower peak. Given what is known about the transmissibility and (the relatively long 5–6 days) incubation period of COVID-19,^1^, ^16^ the efficacy of physical distancing in reducing these important attributes of any epidemic are no surprise.

In the analysis, we have varied the basic reproduction number, the average duration of infections, the initial proportion of cases infected, the susceptibility of children, and the role of younger individuals in transmission dynamics of COVID-19.

In conclusion, non-pharmaceutical interventions based on sustained physical distancing have a strong potential to reduce the magnitude of the epidemic peak of COVID-19 and lead to a smaller number of overall cases. Lowering and flattening of the epidemic peak is particularly important, as this reduces the acute pressure on the health-care system. Premature and sudden lifting of interventions could lead to an earlier secondary peak, which could be flattened by relaxing the interventions gradually.

**This online publication has been corrected. The corrected version first appeared at thelancet.com on/publichealth May 4, 2020**

## Data sharing

Access to computer code is provided in the appendix (p 1). Data used in this study are freely available to the scientific community.
