# Supplementary material for: The effect of control strategies to reduce social mixing on outcomes of the COVID-19 epidemic in Wuhan, China: a modelling study
Source: Lancet Public Health. 2020 Mar 25;5(5):e261–70. doi: 10.1016/S2468-2667(20)30073-6 (PMC7158905; doi:10.1016/S2468-2667(20)30073-6)
Supplement: Supplementary appendix [file mmc1.pdf]

THE LANCET
Public Health

Supplementary appendix

This appendix formed part of the original submission and has been peer reviewed. We post it as supplied by the authors.

Supplement to: Prem K, Liu Y, Russell TW, et al. The effect of control strategies to reduce social mixing on outcomes of the COVID-19 epidemic in Wuhan, China: a modelling study. *Lancet Public Health* 2020; published online March 25. https://doi.org/10.1016/S2468-2667(20)30073-6.

Supplementary Materials

The data and codes can be found at: https://github.com/kieshaprem/covid19-agestructureSEIR-wuhan-social-distancing

As the population contact matrix is related to the next generation matrix (the dominant eigenvalue of which is equal to R_0), reduction in the amount of contact in the population translates to reduction in R_0, so we would expect social distancing measures to be effective.

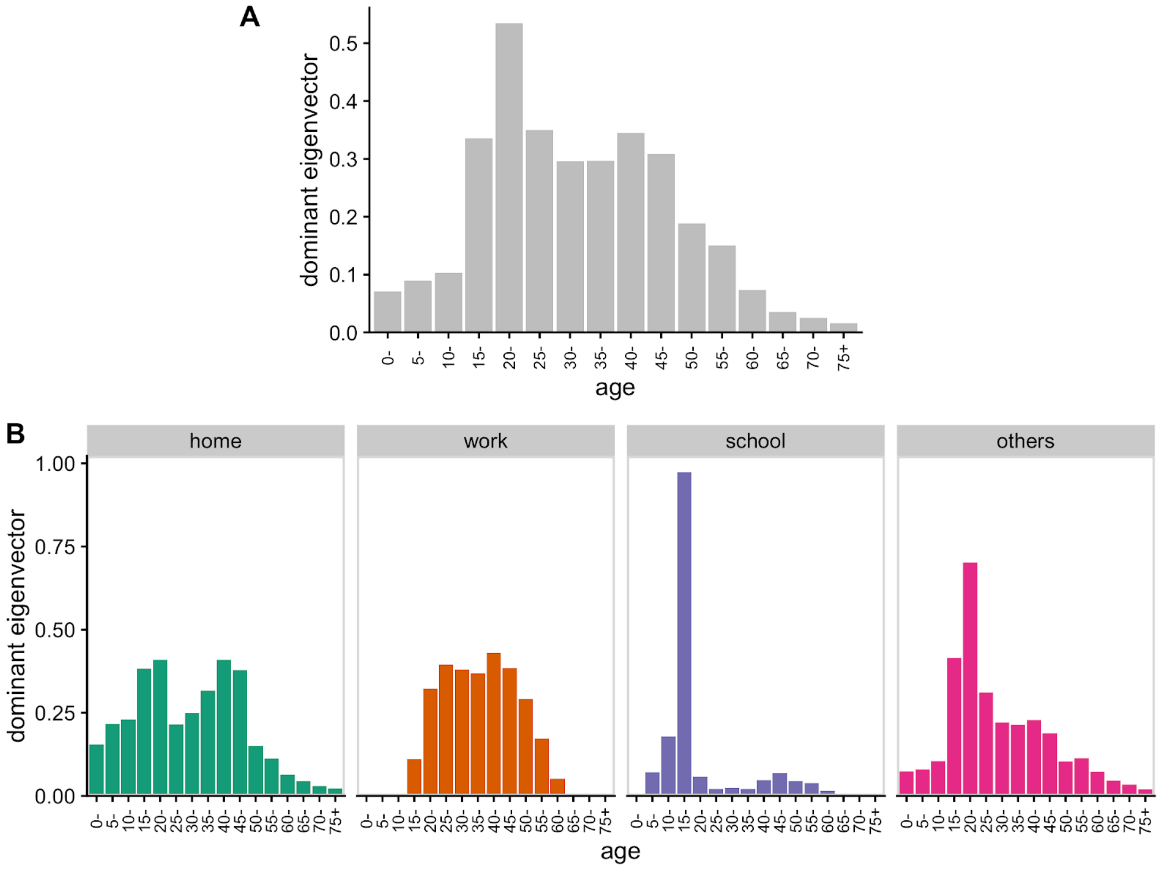

Figure S1. Dominant eigenvectors of synthetic contact mixing matrices for China scaled to Wuhan population size. (A) Dominant eigenvector of the contact matrix including all contacts, (B) eigenvectors of contact matrices by location of contact.

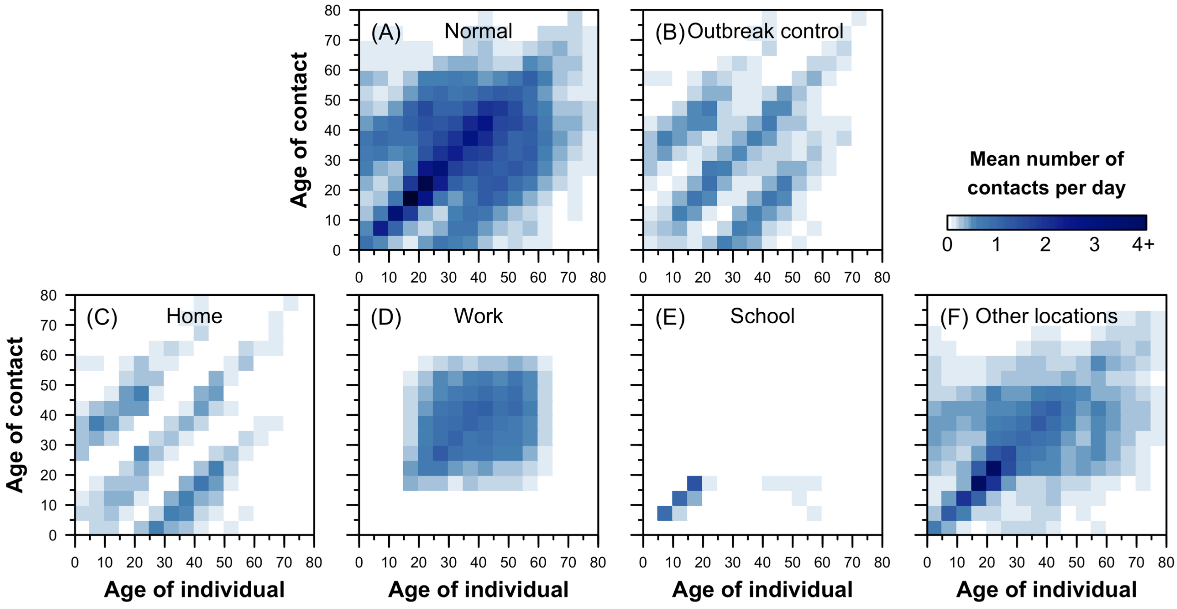

Figure S2. Synthetic age- and location-specific contact matrices for China under various social distancing scenarios (A) before and (B) during the outbreak. The synthetic age-specific contact patterns at home, at the workplace, in school, and at other locations under normal circumstances (i.e. no intervention) are presented in panels C–F. Darker colour intensities indicate higher proclivity of making the age-specific contact.

To estimate the early dynamics of disease transmission in Wuhan, Kucharski and colleagues[1] fitted a stochastic transmission model to multiple datasets on the timing of cases in Wuhan and timing of international exported cases from Wuhan. To ensure that the model was producing plausible results, it was validated using another dataset—confirmed cases in Wuhan from 16th January to 12th February—that was not used in the inference. In addition, the model incorporated both reporting delays and location-specific differences. These estimates of R0 for Wuhan shown in Fig S3 were used to inform the current analysis on human-to-human transmission in Wuhan during the early stages of the outbreak.

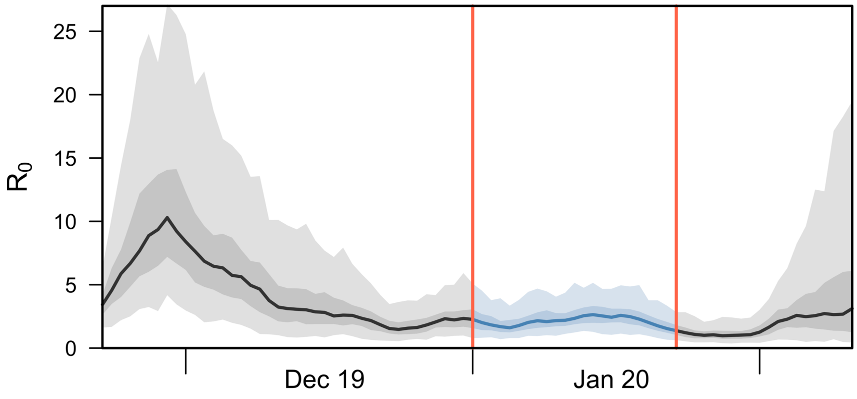

Figure S3. Sampling of the R0 distribution.

[1]Kucharski AJ, Russell TW, Diamond C, *et al.* Early dynamics of transmission and control of COVID-19: a mathematical modelling study. *Lancet Infect Dis* 2020; **0**. DOI:10.1016/S1473-3099(20)30144-4.

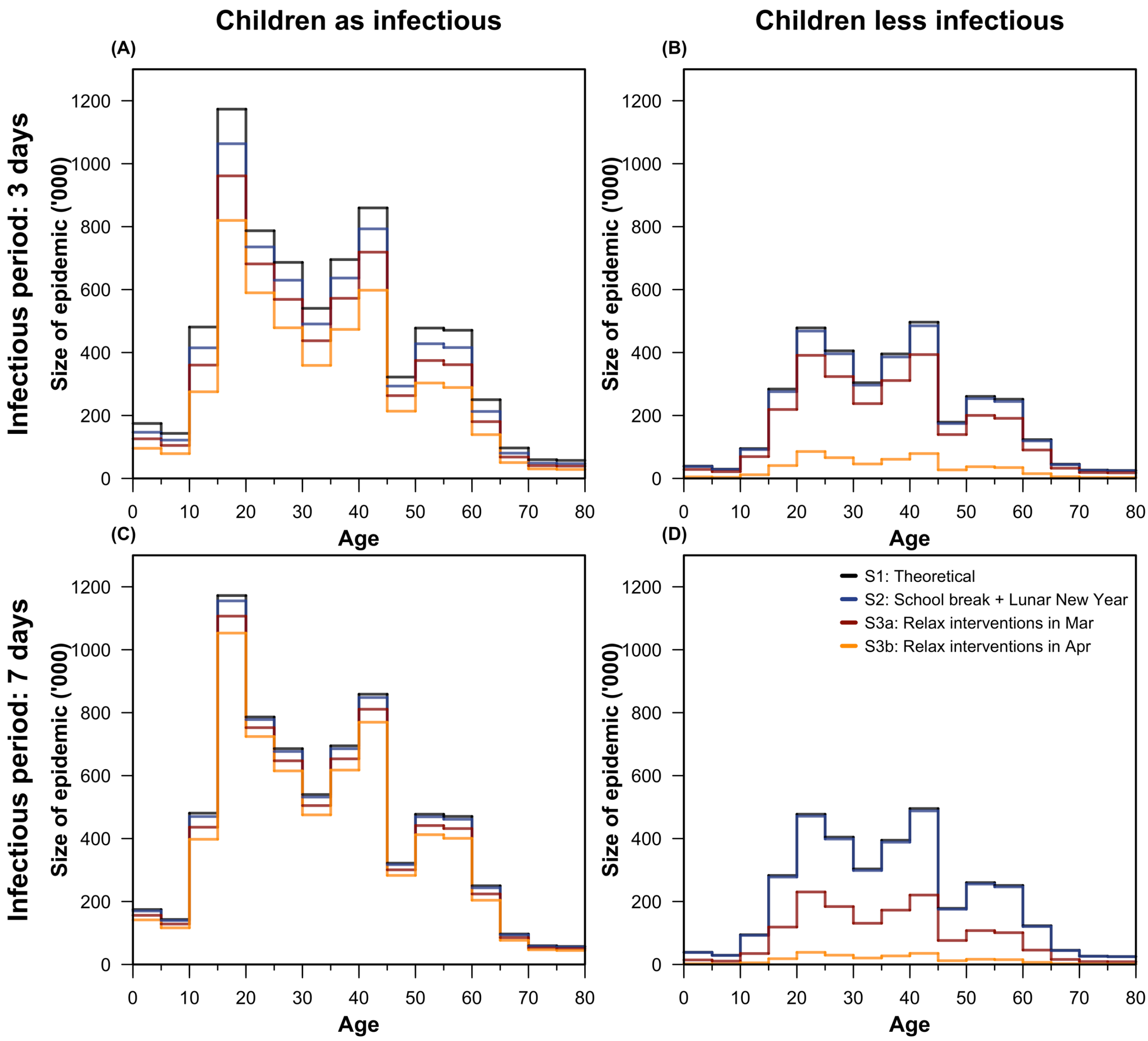

Figure S4. Modelled number of infections in mid-2020 by age for the different social distancing measures, assuming the duration of infectiousness to be (A and B) 3 days and (C and D) 7 days. The total number of cases by age are presented across age and by the different social distancing measures: theoretical no intervention (black), school break and Lunar New Year (blue), intense control measures that are relaxed in a staggered fashion at the beginning of March (red), and intense control measures that are relaxed in a staggered fashion at the beginning of April (orange).

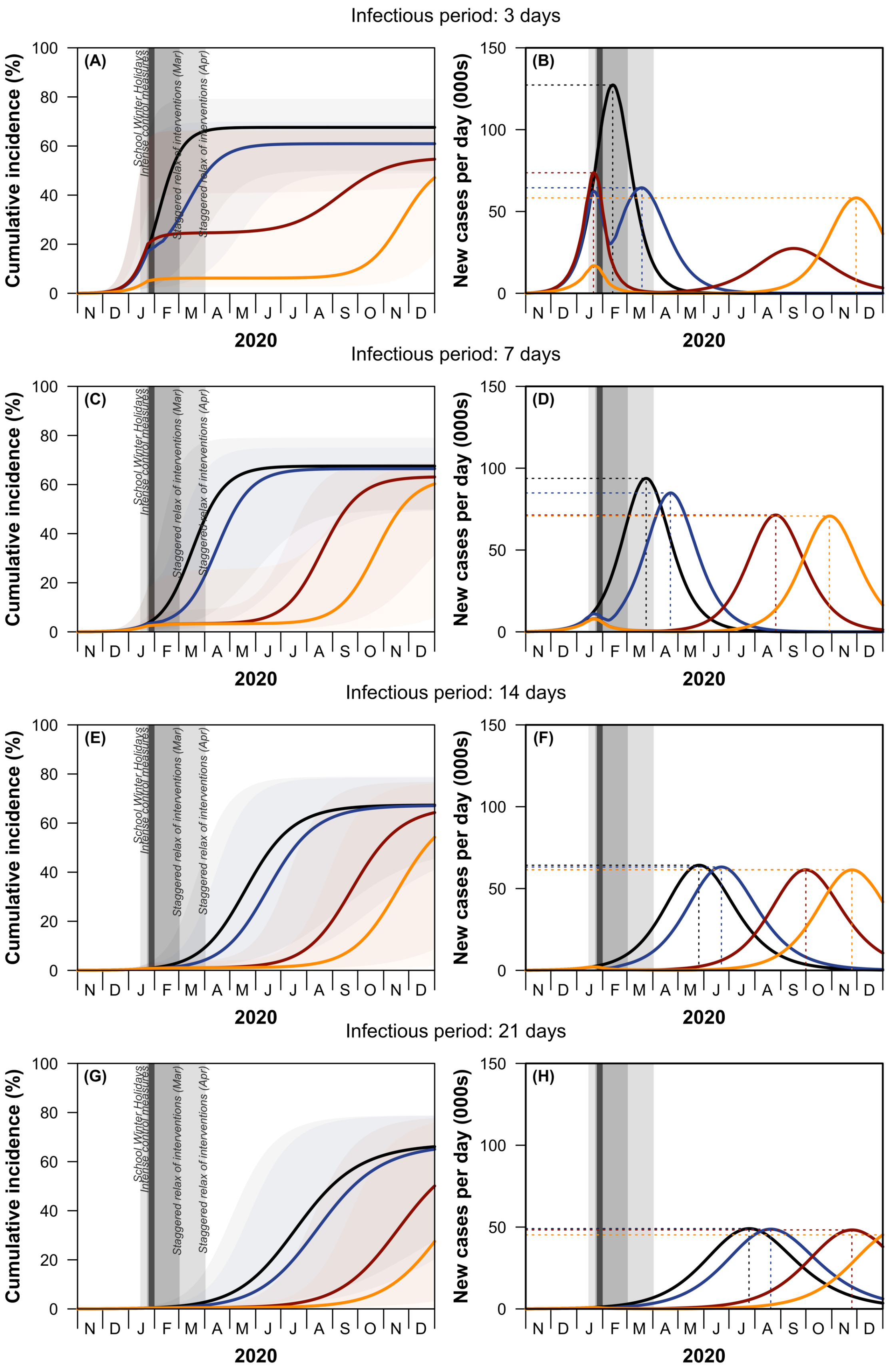

Figure S5. Effects of the different social distancing measures on the cumulative incidence and new cases per day for different duration of infectiousness from late 2019 to end-2020. In the simulation runs, the average duration of infectiousness was assumed to be 3, 7, 14, or 21 days. The median

cumulative incidence, incident cases per day and age-specific incidence per day are represented as solid lines. The 25th and 75th percentile outbreaks are represented by the shaded area in the cumulative incidence. Theoretical no intervention (black line), school break and Lunar New Year (blue line) and intense control measures that are relaxed in a staggered fashion at the beginning of March (red line), and intense control measured that are relaxed in a staggered fashion at the beginning of April (orange line). Shading indicates the timing of the school holidays, Lunar New Year weekend (dark vertical line), intense control measures (dark grey), and staggered return to work followed by school opening (lighter grey).

Conventionally, the transmissibility of a virus is positively associated with viral shedding which is then correlated with symptoms. Much remains still unknown about effects of viral shedding of the novel coronavirus in asymptomatic children, they are inherently difficult to estimate without data from high-quality contact tracing or population serology studies. Having said that, there could be a possibility that subclinical (or asymptomatic) children are more infectious: we simulated this event where subclinical (or asymptomatic) children are *five* times more infectious than subclinical adults, presented in the figure below.

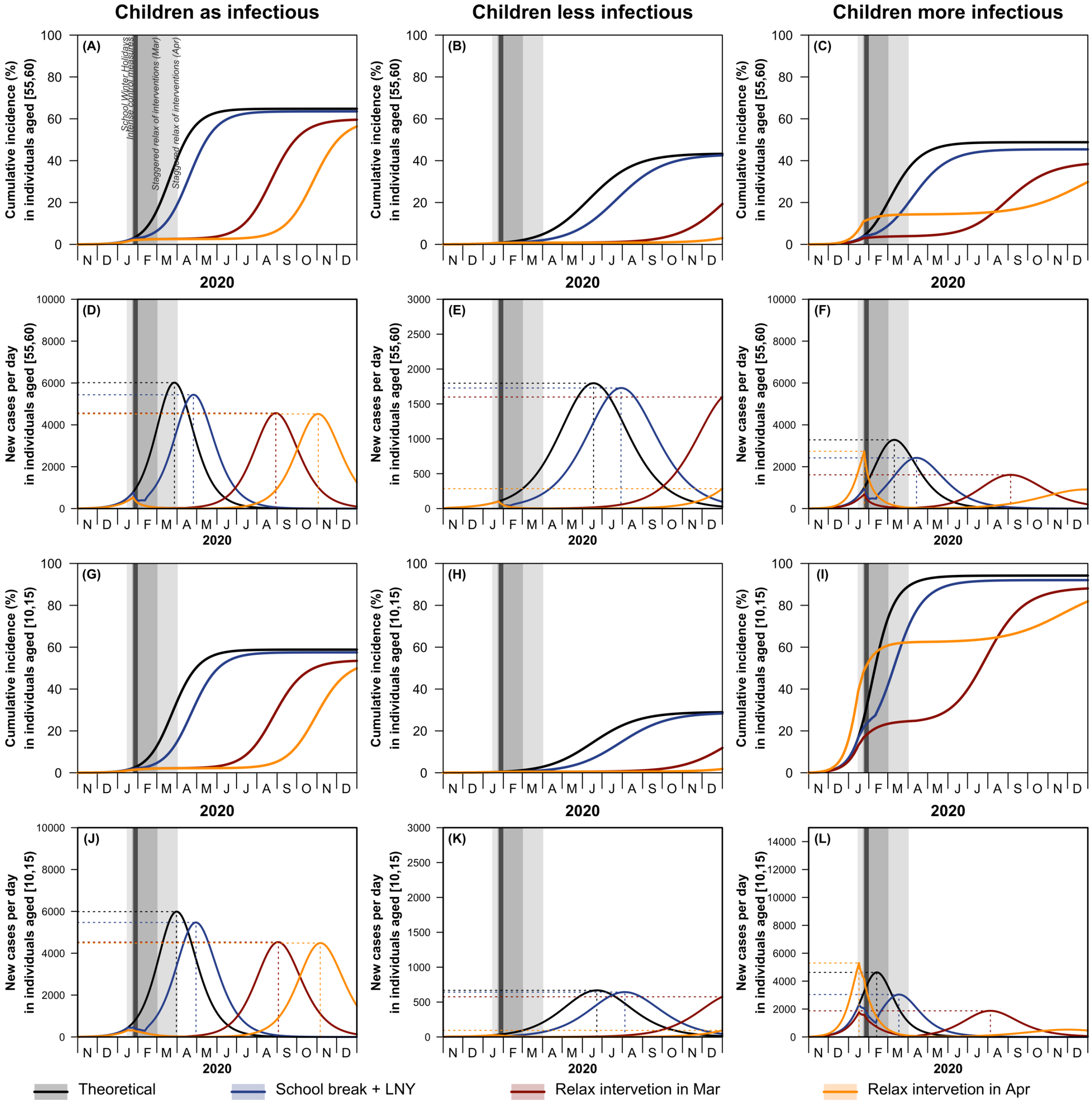

Figure S6. Effects of different intervention strategies on the cumulative incidence and new cases per day among individuals aged 55–60 (A to F) and 10–15 (G to L) from late 2019 to end-2020. Under three scenarios children being equally infectious and children being less infectious and children being more infectious, the effects of social distancing measures were investigated. Theoretical no intervention (black line), school break and LNY (blue line) and intense control measures that are relaxed in a staggered fashion at the beginning of March (red line), and intense control measures that are relaxed in a staggered fashion at the beginning of April (orange line). Shading indicates the timing of the school holidays, Lunar New Year (dark vertical line), intense control measures (dark grey), and staggered return to work followed by school opening (lighter grey).
